# Population pharmacokinetics and optimization of the dosing regimen of digoxin in adult patients

**DOI:** 10.1186/s40780-015-0023-6

**Published:** 2015-09-25

**Authors:** Toshiaki Komatsu, Mami Morita, Futaba Miyaji, Takayuki Inomata, Junya Ako, Koichiro Atsuda

**Affiliations:** Department of Pharmacy, Kitasato University Hospital, 1-15-1 Kitasato, Minami-Ku, Sagamihara, Kanagawa 252-0375 Japan; Department of Cardiovascular Medicine, Kitasato University School of Medicine, Sagamihara, Japan

**Keywords:** Digoxin, Population pharmacokinetics, Dosage regimen

## Abstract

**Background:**

This study aimed to evaluate the population pharmacokinetics of digoxin in Japanese patients and establish a dosage regimen based on the pharmacokinetic data.

**Methods:**

We analyzed 287 serum digoxin samples from 192 individuals by using the nonlinear mixed effects model. We used simulations to optimize the dosage regimen of digoxin to achieve a high likelihood of the target concentration (0.5–0.8 ng/mL).

**Results:**

The total body clearance (CL/F ([L/h]) was calculated using the following formula: CL/F = (1.21 + 0.0532 × CLcr [(mL/min]) × (1 + 0.787 × AMD), where CLcr is the creatinine clearance and AMD is 0 in the case of concomitant administration of amiodarone and 1 otherwise. To achieve the target concentration (0.5–0.8 ng/mL), the dosage of digoxin was 0.0625 mg/day (CLcr < 35 mL/min and AMD = 0); 0.125 mg/day (CLcr, 35–65 mL/min and AMD = 0); 0.1875 mg/day (CLcr, 65–100 mL/min and AMD = 0); 0.0625 mg/every other day (CLcr < 30 mL/min and AMD = 1); and 0.0625 mg/day (CLcr, 30–85 mL/min and AMD = 1).

**Conclusions:**

Our findings suggest that population parameters are useful for evaluating digoxin pharmacokinetics.

## Background

Digoxin is widely prescribed for the treatment of congestive heart failure and atrial fibrillation. Therapeutic drug monitoring of digoxin is recommended because of its narrow therapeutic range [1, 2]. Previous studies have reported several equations and nomograms to enable physicians to determine the appropriate dosage of digoxin for individual patients [3, 4]. In addition, population pharmacokinetic data indicate that clearance of digoxin is influenced by demographic variables such as age, total body weight, and serum creatinine levels [5–7]. However, population pharmacokinetics have not been studied thus far to evaluate the influence of concomitant administration of drugs such as amiodarone, verapamil, and tolvaptan on relative digoxin clearance. Recently, the therapeutic range for digoxin in patients with heart failure with a normal sinus rhythm has been revised to a low and narrow range (0.5–0.8 ng/mL) based on the findings from the digitalis investigation group trial [8]. In this study, we aimed to analyze the population pharmacokinetics of digoxin in the presence of concomitant administration of other drugs. Furthermore, we determined initial dosing regimens to achieve concentrations (0.5–0.8 ng/mL) according to our population pharmacokinetic data.

## Methods

### Ethics statement

Blood samples were collected as part of the routine patient care for therapeutic drug monitoring and laboratory testing when we collect blood samples from patients. This study was approved by the Ethics Committee of Kitasato University Hospital (B13-99: approved on July 24, 2013).

### Data source

Routine clinical pharmacokinetic data (287 observations) were retrospectively collected from 192 adult patients who were administered digoxin (Digosin®; Chugai Pharmaceutical Co. Ltd, Tokyo, Japan or Digoxin-KY®; Toaeiyo Pharmaceutical Co Ltd, Tokyo, Japan) at Kitasato University Hospital between November 2011 and January 2013. The clinical characteristics of patients in this study were shown in Table 1. The patients were inpatients and outpatients. Inpatients were under the supervision of medical and nursing staff. As for outpatients, we checked their doctor’s or nurse’s medical records for patients’ compliance and digoxin trough concentration samples. If this information wasn’t written in medical records, the patient was excluded. Compliance was standardized, and digoxin concentrations were determined. Blood samples were obtained before the administration of the medication. Patient information is provided in Table 1. The collected data included age, gender, height, body weight (BW), serum creatinine level, creatinine clearance (CLcr), ejection fraction (EF), serum potassium level, concomitant medication, and serum concentration of digoxin. Concomitant medication xadministrated in the previously reported to influence digoxin pharmacokinetics were investigated. CLcr was estimated from the serum creatinine level using the Cockcroft-Gault method [9]. One week after administration of digoxin, steady-state concentrations were achieved; subsequently, we drew blood samples before the morning dosing for the assay. The concentration of digoxin was measured using a cloned enzyme immunoassay. The minimum detectable concentration for digoxin was 0.2 ng/mL. The coefficients of variation of both intra- and inter-assay precision were less than 10 %. The present study excluded patients who had any major disorders of the hepatic, gastrointestinal, dialysis or rapidly deteriorating renal function.

**Table 1:** Patient characteristics

Number of Patients	192
Gender (Male:Female)	121:71
EF (%) (>=40: <40)	156:36
Age (year)	71±12^*^
CLcr (mL/min)	56.17±33.76^*^
Weight (kg)	55.47±11.94^*^
Observation	287
0.125 mg /3 days	7
0.125 mg /2 days	17
0.0625 mg /day	14
0.125 mg/day	200
0.25 mg/day	49
Digoxin concentration (ng/mL)	0.90±0.56^*^
Combination medication	
Amiodarone	15
Amlodipine	21
Atorvastatin	14
Azelnidipine	13
Bisoprolol	28
Carvedilol	53
Nifedipine	13
Spironolactone	35
Tolvaptan	8
Antiarrhythmic agent; Class I	12
(Aprindine, Cibenzoline, Flecainide, Pilsicainde, Procainamide)	
Antiarrhythmic agent;Class IV	31
(Bepridil, Diltiazem, Verapamil)	

*Mean ± standerd deviation

EF; Ejunction function, CLcr; Creatine clearance

### Pharmacokinetics model

Data analysis was performed using the nonlinear mixed effects model (NONMEM) program (version VI, level 1.0) developed by Beal and Sheiner [10]. Because the trough serum concentration of digoxin achieved steady-state, we used a simple pharmacokinetic model as follows: Cssij = Dij/(CLij τij), where Cssi is the steady-state serum digoxin concentration measured in the jth patient while he or she received the ith dosage, Dij is the dosage of digoxin in the jth patient, CLij is the total body clearance of digoxin in the jth patient, and τij is the dosing interval for the ith dosage in the jth patient. We used the first-order conditional estimation method (FOCE) for modeling. The inter-individual variability of the parameters was assessed using an exponential error model: Pi = TV(Pi) × exp(ηi), where Pi indicated the individual value, TV(Pi) was the population value for the parameters described in the equation, and ηi was the random deviation of Pi from TV(Pi). The value of ηi was assumed to be independently and normally distributed with a mean of 0 and a variance of ω2. The residual (intra-individual) variability of the parameters was assessed using a proportional error model: Cobs,ij = Cpred,ij × (1 + εij), where Cobs,ij and Cpred,ij denote the j^th^ observed and predicted concentrations for the i^th^ subject, respectively, and ε is a random intra-individual error that is normally distributed with a mean of 0 and variance σ2.

### Covariate analysis

We examined the covariance of the variables, including age, CLcr, BW, EF, systolic blood pressure, and concomitant use of drugs to improve the population pharmacokinetic model. The influence of continuous covariates on the pharmacokinetic parameter TV(P) was modeled according to the following equations: TV(P) = θp + θc × (covariance), and TV(P) = θp × θc^(covariance)^. The covariance that showed a correlation with the pharmacokinetic parameters was introduced into the model. The significance of the influence of covariates was evaluated by a change of -2 log likelihood (the minimum value of the objective function: OBJ). An OBJ decrease of more than 3.84 from the basic structural model (*χ*2: degree of freedom = 1, *P* < 0.05) was considered statistically significant during the forward inclusion process. The full model was structured by incorporating the significant covariates, and the final model was developed using a backward elimination method. When one covariate factor was excluded from the full model, an OBJ that increased more than 6.63 from the full model (*χ*2: degree of freedom = 1, *P* < 0.01) was considered statistically significant.

### Model evolution

The adequacy of fitting was examined by plotting the predicted concentration versus the observed concentration, individual predicted concentration after Bayesian step versus observed concentration, and the weighted residual concentration versus the predicted one. The accuracy and robustness of the final model were assessed using the bootstrap method [11]. A bootstrap sample was generated by repeated random sampling of the original data set, and the size of the bootstrap sample was the same as that of the original sample. We reconstructed 200 bootstrap samples, and the final model was determined by repeatedly testing the 200 bootstrap samples. The mean parameter estimates obtained from replication and calculated normally were compared with those obtained from the original data set.

### Determination of the dosing regimen

Simulation of pharmacokinetics was performed to determine the dosing regimen based on our population pharmacokinetics data. Digoxin concentration was simulated for 1000 patients by using the final population pharmacokinetics model.

The probability that the trough digoxin concentration was in the range of 0.5–0.8 ng/mL in the steady state was calculated as the ratio of the number of simulated patients to the total number of patients. The simulation was performed using Microsoft Excel® 2013.

## Results

**Fig. 1:**
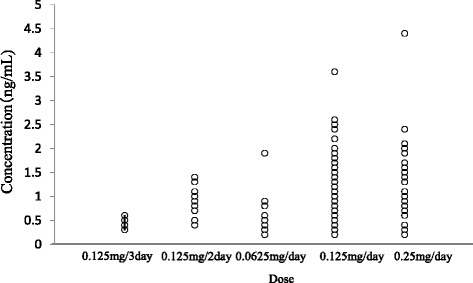
Relationship between the dose of digoxin and serum concentration

The serum concentration of digoxin as a function of the daily dose and each covariate model is shown in Fig. 1 and Table 2, respectively. CLcr, BW, amiodarone, amlodipine, atorvastatin, bisoprolol, carvedilol, and tolvaptan were significant covariates for the CL of digoxin. During the backward deletion from the full model, CLcr and amiodarone remained in the model that caused significant OBJ increase. Therefore, the final model was as follows: CL/F (L/h) = (1.21 + 0.0532 × CLcr [mL/min]) × (1 + 0.787 × AMD), where AMD = 0 for concomitant administration of amiodarone or 1 otherwise.

**Table 2:** Hypothesis testing for fixed effect model of digoxin pharmacokinetics

Fixed model	OBJ	LLD	*p*-value
CL=θ1	−128.969		
θ1+θ2×CLcr	−331.151	202.182	<0.001
θ1+θ2×BW	−139.203	10.234	<0.002
θ1×θ2^Ejection function^ (EF>41=1,EF<40=0)	−129.247	0.278	N.S.
θ1×θ2^Age^ (Age>65=0,Age<64=1)	−131.508	2.539	N.S.
θ1×θ2^Drug^ (0;Cocomitant adminstaraion,1;otherwise)			
Amiodarone	−163.260	34.291	<0.001
Amlodipine	−142.552	13.583	<0.001
Atorvastatin	−135.985	7.016	<0.01
Azelnidipine	−129.016	0.047	N.S.
Bisoprolol	−145.971	17.002	<0.001
Carvedilol	−134.190	5.221	<0.05
Nifedipine	−129.355	0.386	N.S.
Spironolactone	−132.177	3.208	N.S.
Tolvaptan	−133.804	4.835	<0.05
Antiarrhythmic agent;Class I (Aprindine,Cibenzoline, Flecainide,Pilsicainde,Procainamide)	−131.664	2.695	N.S.
Antiarrhythmic agent;Class IV (Bepridil,Diltiazem,Verapamil)	−131.559	2.590	N.S.

*OBJ* the minimum value of objective function, *LLD*-2 log-likefood difference, *N.S* not significant

**Fig. 2:**
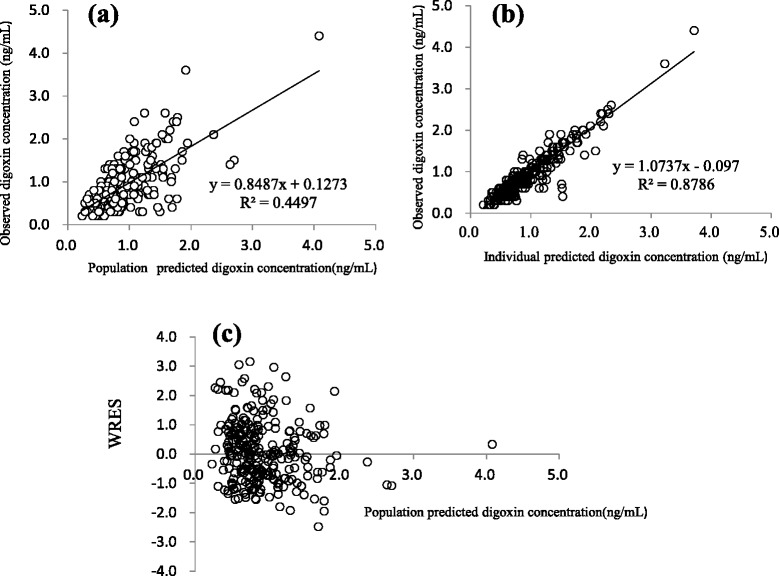
**a** Population predicted concentrations from the final model; (**b**) Individual predicted concentrations from the final model. **c** Individual predicted concentrations from the final model. Scatter plot of weighted residuals (WRES) versus predicted concentration

The coefficient of variation (CV) of the inter-individual variability (ω^2^) of CL and the residual variability (σ^2^) were 32.2 and 25.5 %, respectively (Table 3). Assessment of the predictive performance of the final model is presented in scatter plots of the observed concentration versus the population-predicted concentration (Fig. 2a) and the individual-predicted concentrations of digoxin (Fig. 2b). Weighted-residual concentration versus the population predicted concentration is shown in Fig. 2c. The plots were symmetrically distributed around the line of identity, which indicated that the model adequately described the serum concentration of digoxin. In the bootstrapping analysis of the final model, 180 of 200 showed successful results and the values of parameters used in the final model generated from the bootstrap analysis were similar to those of the developed model (Table 4).

**Table 3:** Final estimates for the population pharmacokinetic parameters of digoxin

Population mean parameters	
	CL(L/h)= (1.21+0.0532×CLcr(mL/min)) ×(1+0.787×AMD)
	(AMD = 0 for cocomitant adminstarion of amiodarone, otherwise 1)
Interindividual variance	
	ω(CL)=32.2 %
Intraindividual variance	
	σ=25.5 %

**Table 4:** Results of bootstrap validation

Parameter	Final Model^a^	Bootstrap^b^	95% Confidence interval
	Mean ± S.E.	Mean ± S.E.	[Lower, Upper]
θ1	1.21 ± 0.21	1.30 ± 0.05	[1.20-1.39]
θ2	0.0532 ± 0.0068	0.0543 ± 0.0016	[0.050-0.057]
θ3	0.787 ± 0.187	0.803 ± 0.018	[0.768-0.838]
ωCL	0.104 ± 0.017	0.324 ± 0.006	[0.312-0.336]
σ	0.065 ± 0.010	0.340 ± 0.086	[0.171-0.509]

CL=(θ1+θ2×CLcr)×(1+θ3×AMD)

^a^ Obtained from the original data set

^b^ Calculated from 200 bootstrap replicates(180 convergence)

**Fig. 3:**
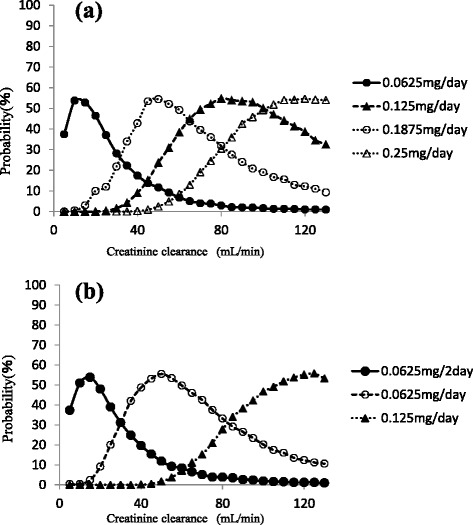
Probability (%) of trough concentration of digoxin being within 0.5–0.8 ng/mL at CLcr ranging from 5 to 130 mL/min with amiodarone (**a**) or without administration (**b**)

**Fig. 4:**
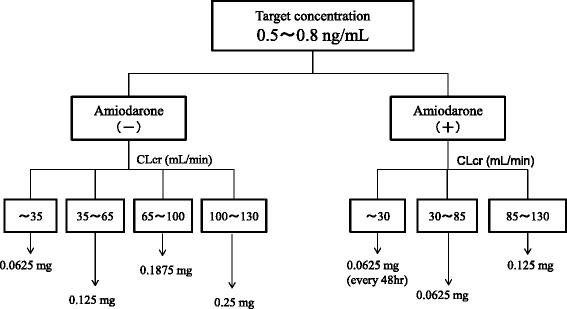
Nomogram for target serum digoxin concentration. The proposed nomograms are presented as a target serum digoxin concentration of 0.5–0.8 ng/mL

We used the final model and performed a simulation to determine the dosing regimen in patients with renal impairment and in those with concomitant administration of amiodarone. Digoxin concentration was simulated for 1000 patients with CLcr ranging from 5 to 130 mL/min with or without amiodarone administration. The simulation was performed for dosages ranging from 0.0625 mg/every 2 days to 0.25 mg/day. The probabilities of trough digoxin concentrations being in the range of 0.5–0.8 ng/mL in the steady state are shown in Fig. 3. The typical initial dose of digoxin at various CLcr with or without amiodarone based on simulation experiments is shown in Fig. 4.

## Discussion

To our knowledge, this is the first study in which a dosing regimen based on population pharmacokinetics has been proposed. We analyzed the sampling data for digoxin obtained from routine clinical data by using the NONMEM. This study focused on how drugs interact with digoxin. Our results showed that amiodarone and CLcr were significant covariants for the systemic clearance of digoxin. The systemic clearance consists of elimination rate constant and distribution volume. However, our model can’t individually assess these parameters because our data is trough concentration sampling. The rate constant is mainly affected by creatinine clearance and amiodarone. Typically, digoxin is thought to be eliminated by the kidney. Additionally, previous studies showed that CLcr is an affective factor [5–7]. Regarding drug interactions, amiodarone, but not amlodipine, atorvastatin, bisoprolol, carvedilol, or tolvaptan, remained during the backward deletion. Beta blockers such as bisoprolol and carvedilol increase the maximum plasma concentration of digoxin by about 1.3-fold [12]. Similarly, administration of 10 mg atorvastatin increases the maximum plasma concentration of digoxin by about 1.2-fold [13]. Administration of 60 mg tolvaptan increases the maximum plasma concentration of digoxin by about 1.3-fold [14]. Schwartz JB reported that amlodipine does not significantly influence steady-state digoxin concentrations [15]. Chen R [5] and Yukawa E [6] showed that spironolactone and calcium channel blockers affect the clearance of digoxin. However these factors result in about a 20 % decrease in digoxin clearance, and also these studies didn’t examine amiodarone. Therefore, we concluded that these medications were not included as final factors. Amiodarone increased the trough level of digoxin concentration by approximately two-fold [16]. The main reason that amiodarone increases the serum concentration of digoxin is the inhibition of digoxin secretion from renal tubules and the inhibition of the P-glycoprotein membrane transporter system [17]. Our results showed that the population means showed a good predictive performance. The final model lacked bias, despite the drug concentration and the observed drug concentration being almost identical to the individual predicted concentration after the Bayesian steps. The weighted residuals were acceptable to within three standard deviations, which are generally recognized as criteria for no selection biases. In addition, the convergence ratio on bootstrap data was significantly high (Table 4). Thus, the robustness of the model was sufficiently confirmed. The difference between θ of the final model estimate and that of the bootstrap means was relatively small. Therefore, we concluded that the final model had a good predictive performance. In future studies, we will perform external validation for our new population pharmacokinetics model. The probability of the trough concentration of digoxin being in the therapeutic range of 0.5–0.8 ng/mL for congestive heart failure is shown in Fig. 3. This regimen suggests the typical initial dosage of digoxin in patients at various CLcr with co-administration of amiodarone. The different initial dosages of digoxin calculated using the Koup [18] and Jusko [19] method depending on different values of CLcr to achieve a target digoxin concentration of 0.7 ng/mL were as follows: CLcr < 30 mL/min, start at 0.0625 mg every day; CLcr 30–80 mL/min, start at 0.125 mg every day; and CLcr > 120 mL/min, start at 0.25 mg every day. In addition, Bauman JL et al [4] reported a nomogram for achieving a steady-state concentration of 0.7 ng/mL on the basis of creatinine clearance and IBW or height. These findings are similar to those obtained using our new dosing regimen without amiodarone. This initial dosage regimen will be useful for reasonable therapies using oral digoxin for congestive heart failure. Some limitations exist in this study. Some studies have shown that the serum concentration of digoxin increases after concomitant administration with other antiarrhythmic drugs such as verapamil and bepridil [20, 21]. We did not include these factors because very few patients in our study received concomitant administration of these drugs. Additionally, we didn’t examine the effect of digoxin transportation role of P-glycoprotein inhibitors such as itraconazole, cyclosporine and clarithromycin [22–24] because these medicines are not administered. Therefore our nomogram is not adapted with concomitant with these medications. Our population model didn’t consider volume of distribution or absorption phase. Therefore our model didn’t simulate these phase. A prospective study using this regimen is necessary to investigate the robustness and reliability of our model.

## Conclusion

Our results suggested that these dosage regimens would provide maximum therapeutic benefit of digoxin, and achieve the overall goal of reducing the toxicity in patients in whom the dosage of digoxin exceeds the therapeutic range.
